# Emergency Surgical Intervention in Microwave Ablation-Induced Massive Lung Necrosis

**DOI:** 10.7759/cureus.54542

**Published:** 2024-02-20

**Authors:** Ali Kimiaei, Seyedehtina Safaei, Pinar Çağan, Cemal Asim Kutlu

**Affiliations:** 1 Thoracic Surgery, Bahçeşehir University, Istanbul, TUR

**Keywords:** thoracotomy, lung tumor, massive lung necrosis, pulmonary metastasis, microwave ablation

## Abstract

Microwave ablation (MWA) has become an increasingly used procedure for the management of lung nodules in recent years. Here, we report a 33-year-old female presenting with massive pulmonary necrosis and tension pneumothorax after MWA for metastatic colon cancer. She required surgical intervention, including thoracotomy, debridement, and wedge resection, for the management of these complications.

## Introduction

There are several indications for Microwave Ablation (MWA) in the management of lung cancer. These include both primary and secondary lung tumors, particularly for patients with up to five pulmonary metastases <3.5 cm, or primary non-small-cell lung tumors ≤3 cm in diameter [1].

We report a rare complication of MWA in a 33-year-old female with lung metastases from colon cancer. After ablation, massive pulmonary necrosis and tension pneumothorax arose, which were managed by the removal of serous pockets containing hematoma and wedge resection in the lateral basal segment of the lower lobe.

## Case presentation

**Figure 1:**
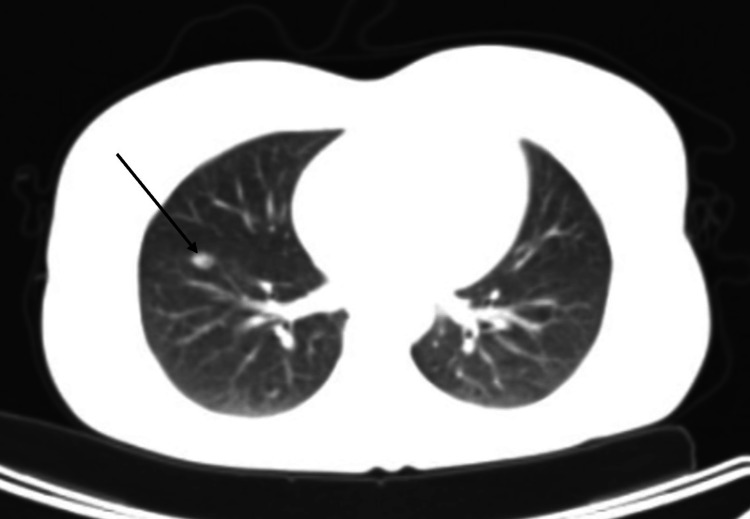
CT scan showing a prominent nodular lesion indicative of metastatic foci (black arrow)

A 33-year-old female patient presented to the emergency department with worsening back pain and dyspnea three days after undergoing MWA for liver and lung metastases. The patient's medical history was significant for metastatic colon cancer, smoking, and alcohol use. Previous surgical interventions include segmental colon and liver resections. The CT scan performed before the procedure showed a metastatic nodule on the right lung near the interlobar fissure and visceral pleura (Figure 1).

The recent MWA procedure employed the Surgnova Dophi 150E ablation device (Zhejiang, China) and was done using CT-guiding at 75W for 5 minutes, creating a 3×3 cm zone. Upon physical examination, decreased breath sounds in the lower right, inspiratory crackles in the lower and middle right, and minimal crackles in the lower left were noted. Eventually, a chest computed tomography (CT) revealed a minimal pneumothorax in the right lung, pleural effusion, and consolidations in the lower right lobe. The patient was admitted, and Tazocin (piperacillin, tazobactam), intravenous fluids, and nasal oxygen (2 L/min) were administered.

**Figure 2:**
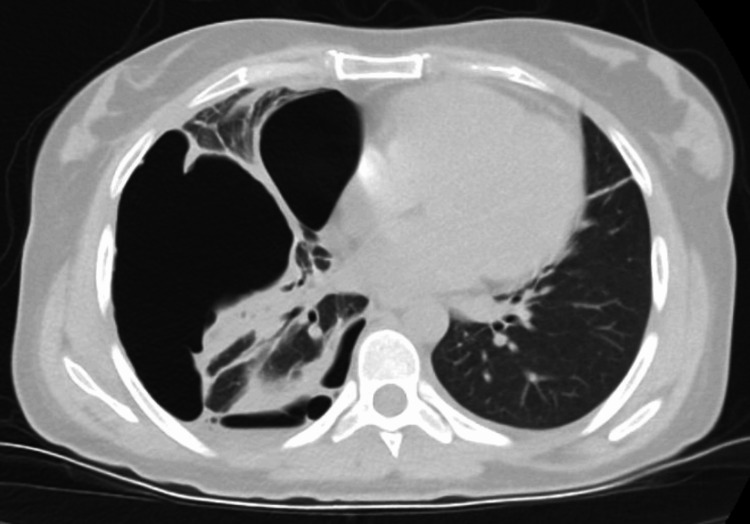
Pulmonary CT angiography showing localized pleurisy on the right, fluid in the right interlobar fissure, and parenchymal collapse in the lower zones of both lungs.

Subsequently, two days later, a chest x-ray revealed an increase in fluid, and the patient developed dyspnea. A catheter thoracostomy was performed, revealing hemorrhagic fluid. Pulmonary computed tomography angiography (CTA) revealed localized pleurisy on the right, minimal pleurisy on the left, fluid in the right interlobar fissure, and parenchymal collapse in the lower zones of both lungs (Figure 2). Due to the hemorrhagic fluid and the radiological findings, thoracoscopic debridement was recommended. However, the patient declined this intervention.

Nine days after the emergency visit, the patient's general condition improved, and the effusions regressed. She was discharged in the morning but returned to the emergency department later that day due to sudden, severe chest pain. A chest x-ray revealed a total pneumothorax, and a tube thoracostomy was applied.

**Figure 3:**
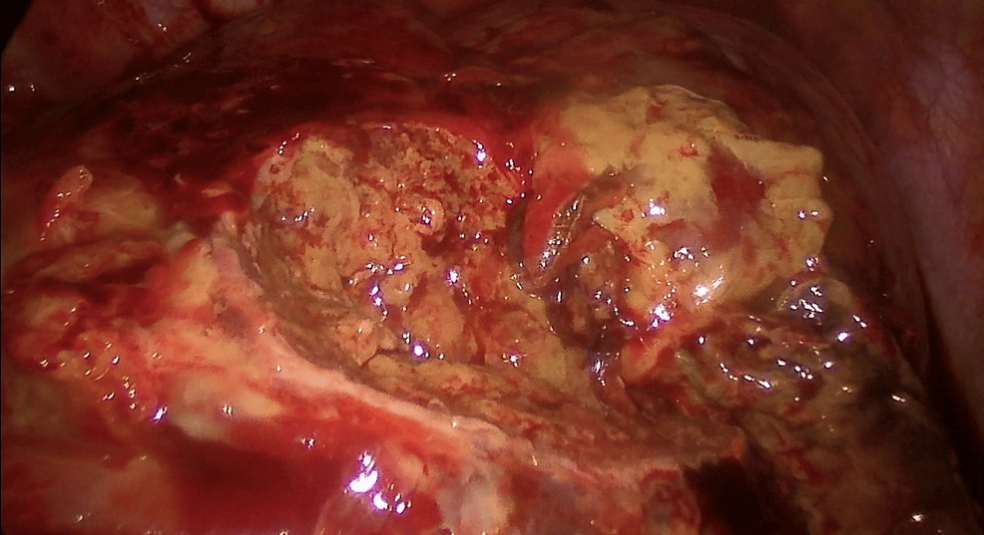
Intraoperative image showing extensive pulmonary necrosis

Afterward, a thoracotomy was performed to control air leakage. With the patient in the left lateral decubitus position, a 3 cm incision was made at the right 6th intercostal space to access the thoracic cavity. Upon optical observation, serous pockets containing hematoma in both anterior and posterior locations were identified. The pockets were opened, and hematoma and fibrin debris were cleared. Following pneumolysis, the upper lobe and the 6th segment were intact, while the lateral segments of the lower lobe and the middle lobe formed a cavity covered in yellow fibrin with visible bronchial openings (Figure 3).

**Figure 4:**
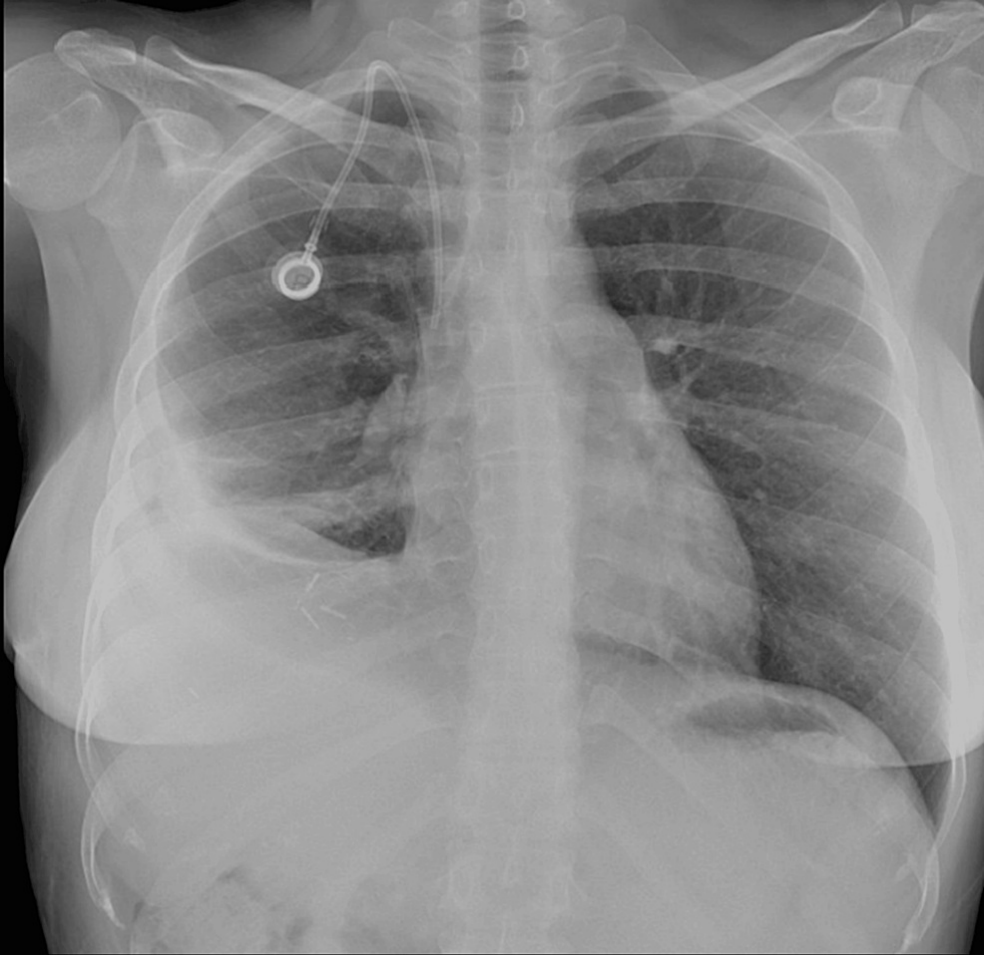
Post-operative chest X-ray showing successful resolution

The damaged parenchyma was approximated with Vicryl sutures, and a wedge resection was performed with three staples to partially close the defect in the lateral basal segment of the lower lobe. Due to the inability to fully close the defect, the incision was enlarged for primary repair with 3/0 Vicryl in the defective areas of the middle and lower lobes. Finally, air leakage control was successful, and following bleeding control, one 24F chest tube was inserted, and the layers were properly closed. The patient successfully recovered and was discharged two days later (Figure 4).

## Discussion

The surgical management of lung metastases is personalized based on the characteristics of the tumor, its extensions, and its stage [2]. Microwave and radiofrequency ablation can be potential alternatives if the tumor is not resectable or in the case of patient refusal.

In a recent meta-analysis on the utilization of MWA in lung cancer, the study reported a complete ablation rate of 81.1% (75.8%-86.0%) [3]. The median local tumor progression-free survival (LTPFS) was 31.5 months (19.0-44.0 months), and the overall survival (OS) was 25.6 months (19.4-31.8 months) [3]. Stratification by tumor type revealed an estimated OS of 18.7 months for pulmonary metastasis and 24.4 months (16.9-31.8 months) for primary lung cancer [3]. Progression-free survival (PFS) was 8.4 months (3.6-13.2 months), with a 3-year PFS rate of 56.0% (41.1%-70.9%) [3].

Furthermore, in a study assessing the safety of MWA procedures adjacent to the interlobar fissure, the overall success rate for ablation was 95.5%, and the PFS rates at 3, 6, 9, and 12 months were 89.4%, 83.3%, 74.2%, and 63.6%, respectively [4]. Common complications observed included pneumothorax (34.8%), pleural effusion (24.2%), cavity formation (18.2%), and pulmonary infection (7.6%) [4]. Other complications of MWA include pulmonary hemorrhage, chest wall injury, pulmonary abscess, thermal injury to adjacent structures, and tumor recurrence [1]. It was suggested that puncturing the antenna through the interlobar fissure might pose a potential risk for complications [4].

The only reported case of massive lung necrosis after MWA involved an elderly male patient in his 80s diagnosed with lung squamous cell carcinoma [5]. On the fourth day post-MWA, the development of pneumothorax and dyspnea and failure to manage the symptoms resulted in an urgent left upper lobectomy [5]. The authors suggested that the elastic properties of the lung tissue in the elderly are more fragile, requiring a reduced frequency and time threshold when performing MWA in this population [5]. However, massive necrosis in our 33-year-old patient raises questions about potential contributing factors in younger individuals, such as tumor location and characteristics or technical aspects of the procedure.

## Conclusions

In conclusion, while MWA can be used in the management of lung metastases, it is not a complication-free procedure, and it is crucial to bear in mind the elevated risks of complications, particularly for lesions proximate to the visceral pleura. We believe that the ablation of lesions in these locations should be carefully assessed and reconsidered, given the increased risk observed in our case and previous studies.
